# Supplementary material for: Increased Replication Stress Determines ATR Inhibitor Sensitivity in Neuroblastoma Cells
Source: Cancers (Basel). 2021 Dec 10;13(24):6215. doi: 10.3390/cancers13246215 (PMC8699051; doi:10.3390/cancers13246215)
Supplement: Supplementary file 1 [file cancers-13-06215-s001.zip › cancers-1476678-supplementary.pdf]

cancers

MDPI

Supplementary Material

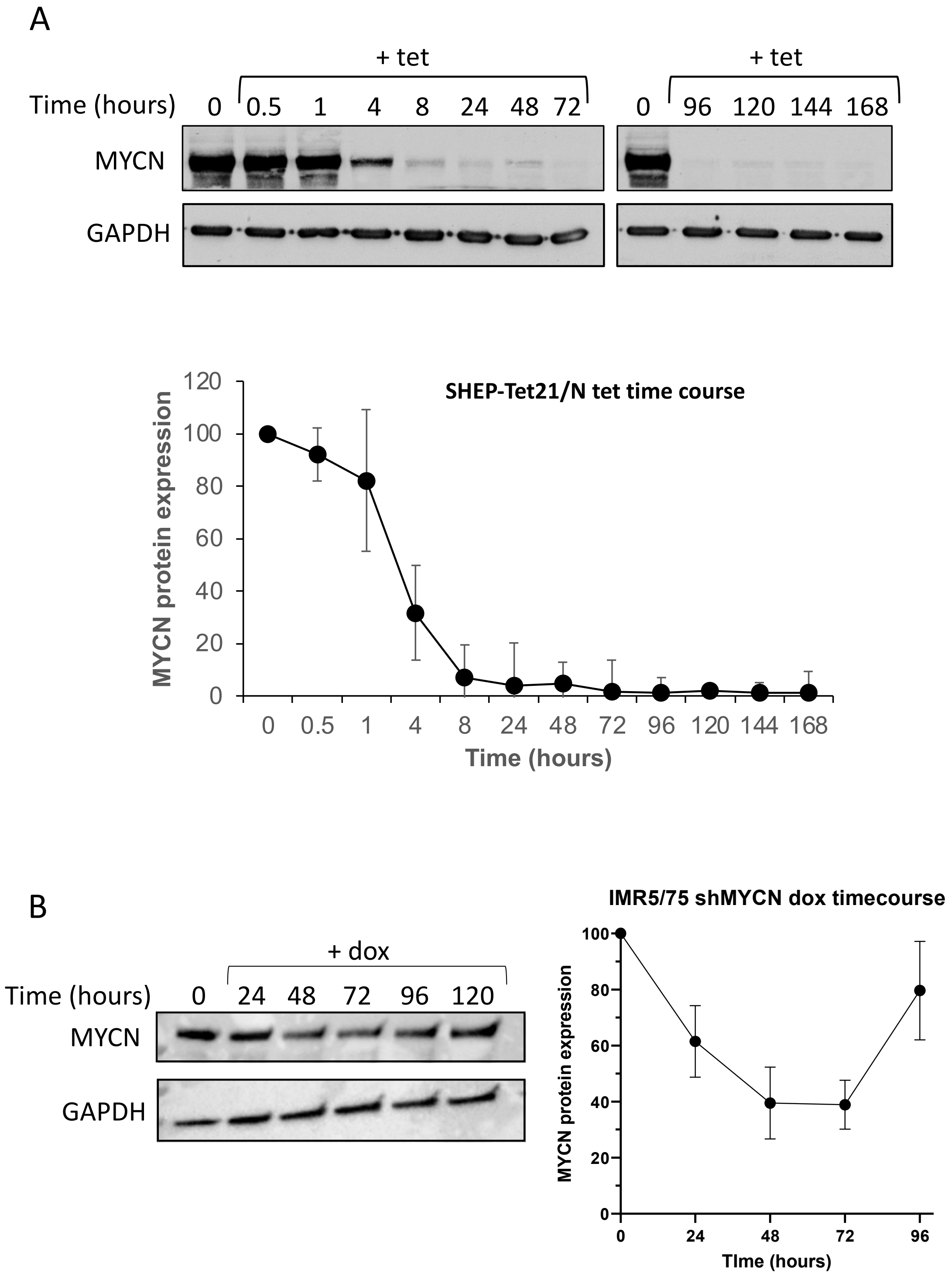

Figure S1. Representative Western blot images with time of MYCN expression in SHEP-Tet21/N cell line and IMR5/75 shMYCN cell line. (A) Representative Western blot image and quantification of MYCN expression of MYCN expression in SHEP-Tet21/N NB cell line in response to 1 μg/mL tetracycline (B) representative Western blot image and quantification of MYCN expression in IMR5/75 shMYCN in response to 120 hours treatment with dox 1 μg/ml over 120 hours (5 days). Data are mean ± SEM for 3 independent experiments. (C) Full western blots and integrated density readings of A and B.

Scheme 1. continued.

C

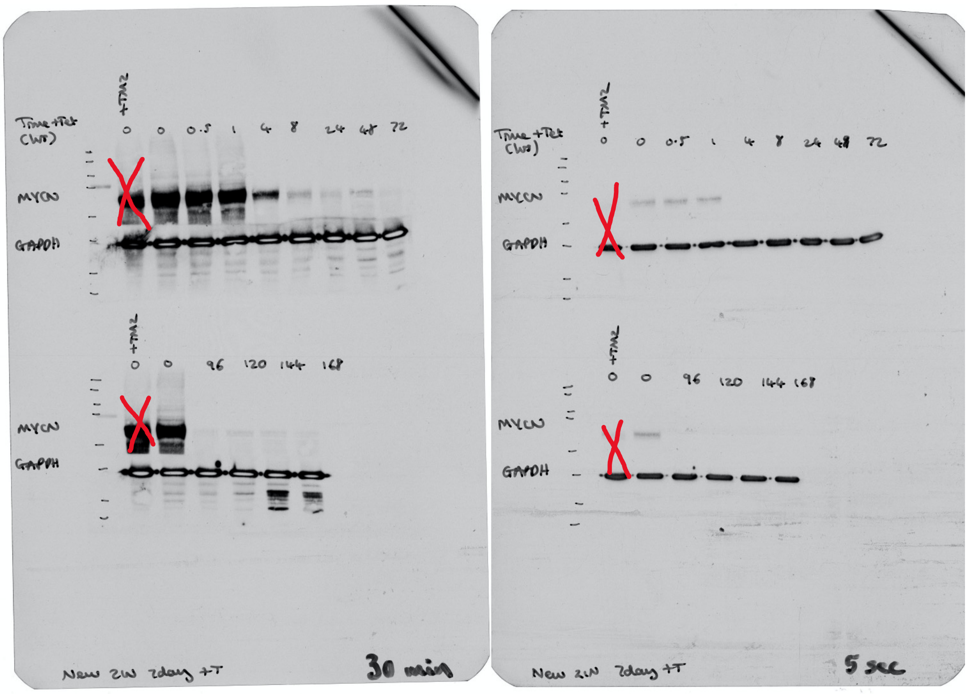

	hours + tet (intergrated density reading)													
	0	0.5	1	4	8	24	48	72		0	96	120	144	168
MYCN	1276776	1177683	1050002	404129	90639	49435	59167	20657		1208196	17816	24745	17869	16304
GAPDH	295987	325393	304599	305762	310151	351796	315159	308674		272661	286172	303959	304535	281742

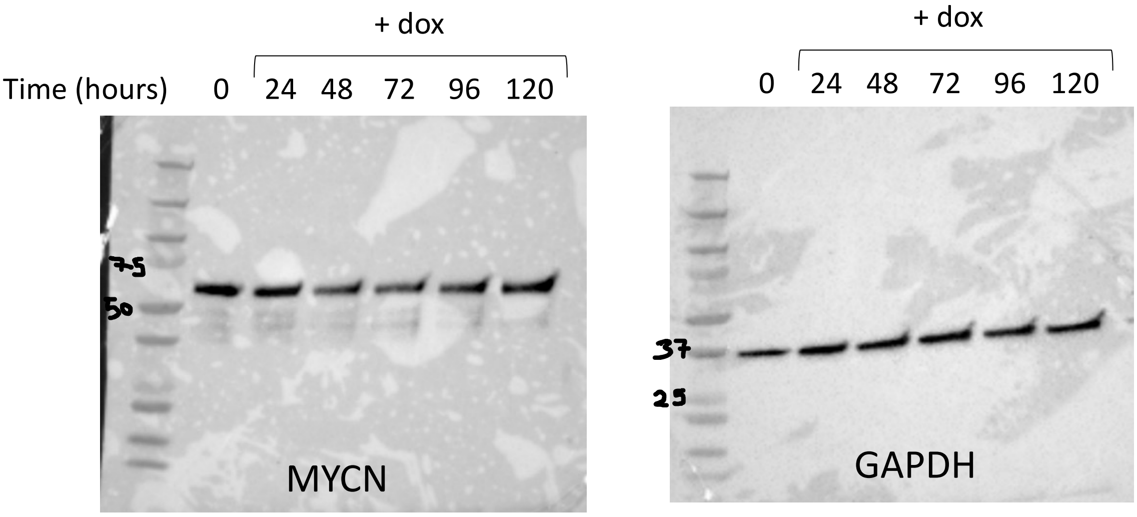

	hours + dox (integrated density reading)					
	0	24	48	72	96	120
MYCN	90804	75483	57694	54262	74533	91033
GAPDH	57257	77427	75569	81745	70966	82363

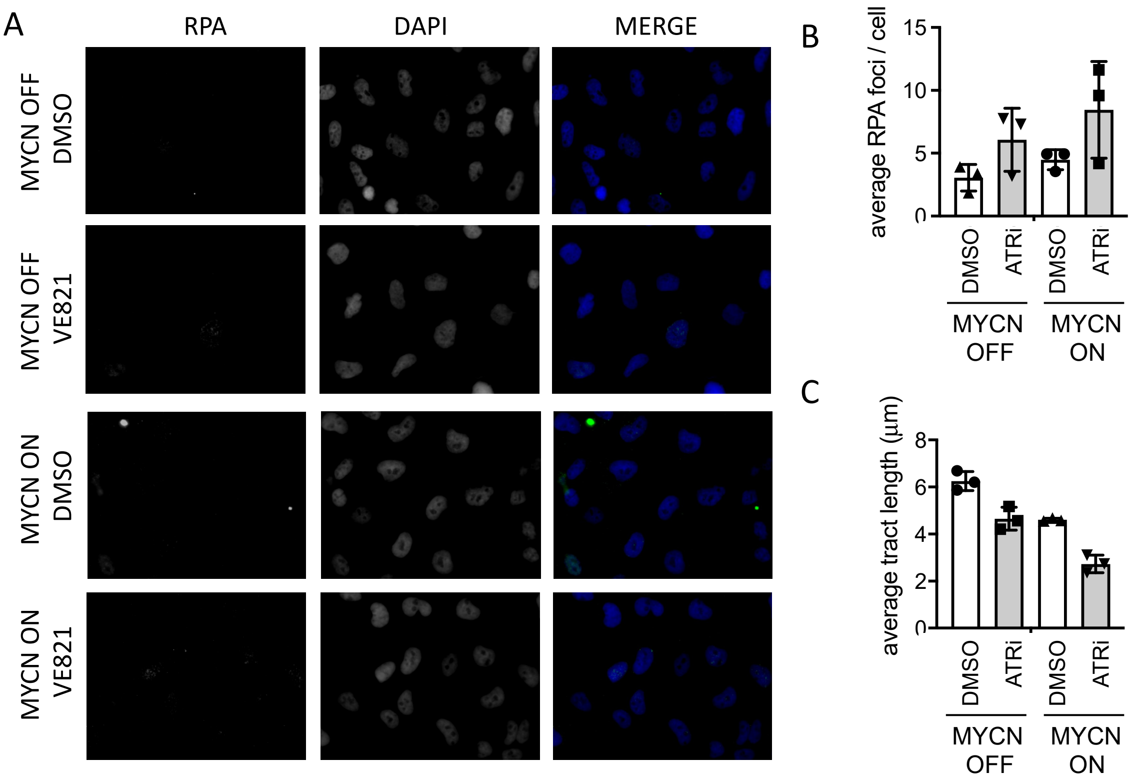

Figure S2. Example images and average Number of RPA foci/cell in SHEP-Tet21/N cells with MYCN ON or MYCN OFF. (A) Example images and (B) average Number of RPA foci/cell in SHEP-Tet21/N cells with MYCN ON or MYCN OFF 24 h post treatment with 1µM VE-821 or DMSO control. Data are independent repeats, pooled data are shown in Figure 2. (C) DNA fibre analysis of replication fork speed and stalling in VE-821 treated SHEP-Tet21/N cells with MYCN ON and MYCN OFF. Cells were incubated in 0.5 µM VE-821 or DMSO control and then pulse labelled with CldU, for 20 min, and labeled switched to IdU for 20 min. Average DNA fibre length (µm) (CIdU), at least 100 forks were counted on each of three separate occasions, means of individual repeats are shown pooled data are shown in Figure 2.

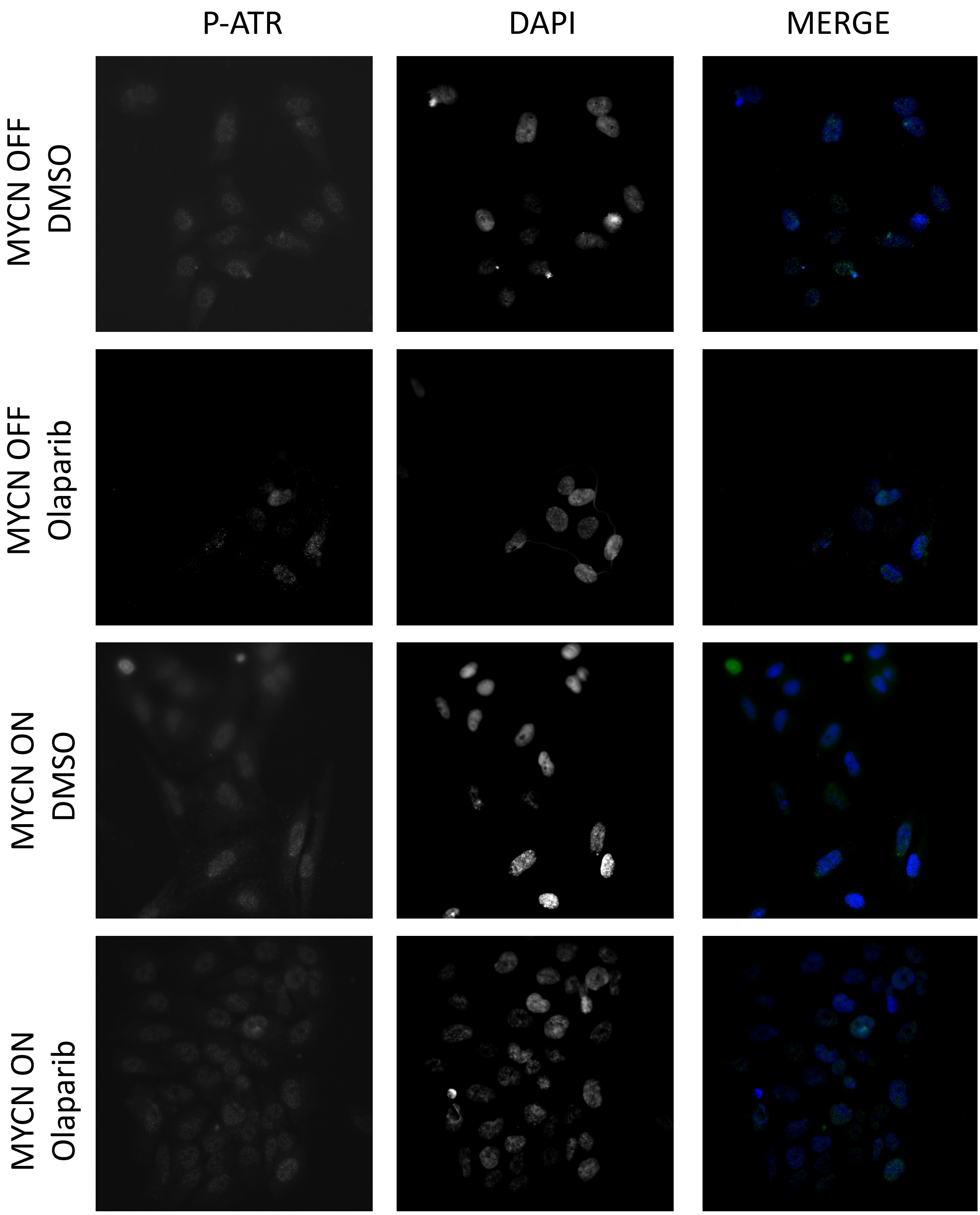

Figure S3. Example p-ATR images stained for phospho-ATR(Thr1989). (A) Example p-ATR images stained for phosph-ATR(Thr1989), DAPI and merge in SHEP-Tet21/N cells with MYCN ON or MYCN OFF 24 h post treatment with 1μM PARP inhibitor olaparib or DMSO control.

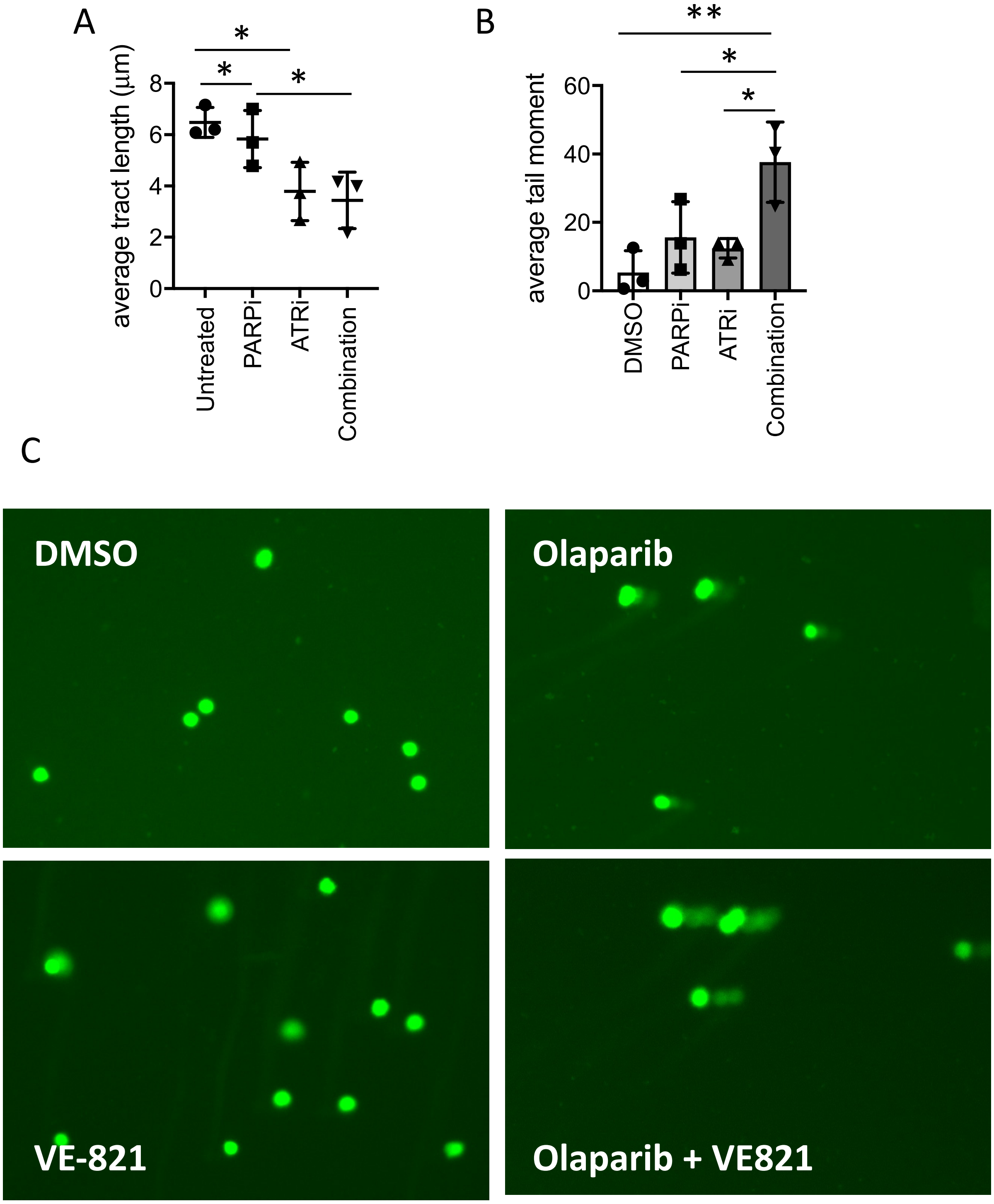

Figure S4. Mean DNA fibre length and COMET tail moment from 3 independent repeats and representative COMET assay images cells. DNA damage; In each experiment cells were incubated in 0.5 μM VE-821 and or 0.5 μM olaparib or DMSO control. Mean DNA fibre length (CIdU - μm) from 3 repeats where pooled data is shown in figure 4. Mean tail moment of individual repeats for alkaline COMET assay where pooled data is shown in figure 4. (**C**) Representative COMET assay images cells are stained with SYBR Safe DNA gel stain.

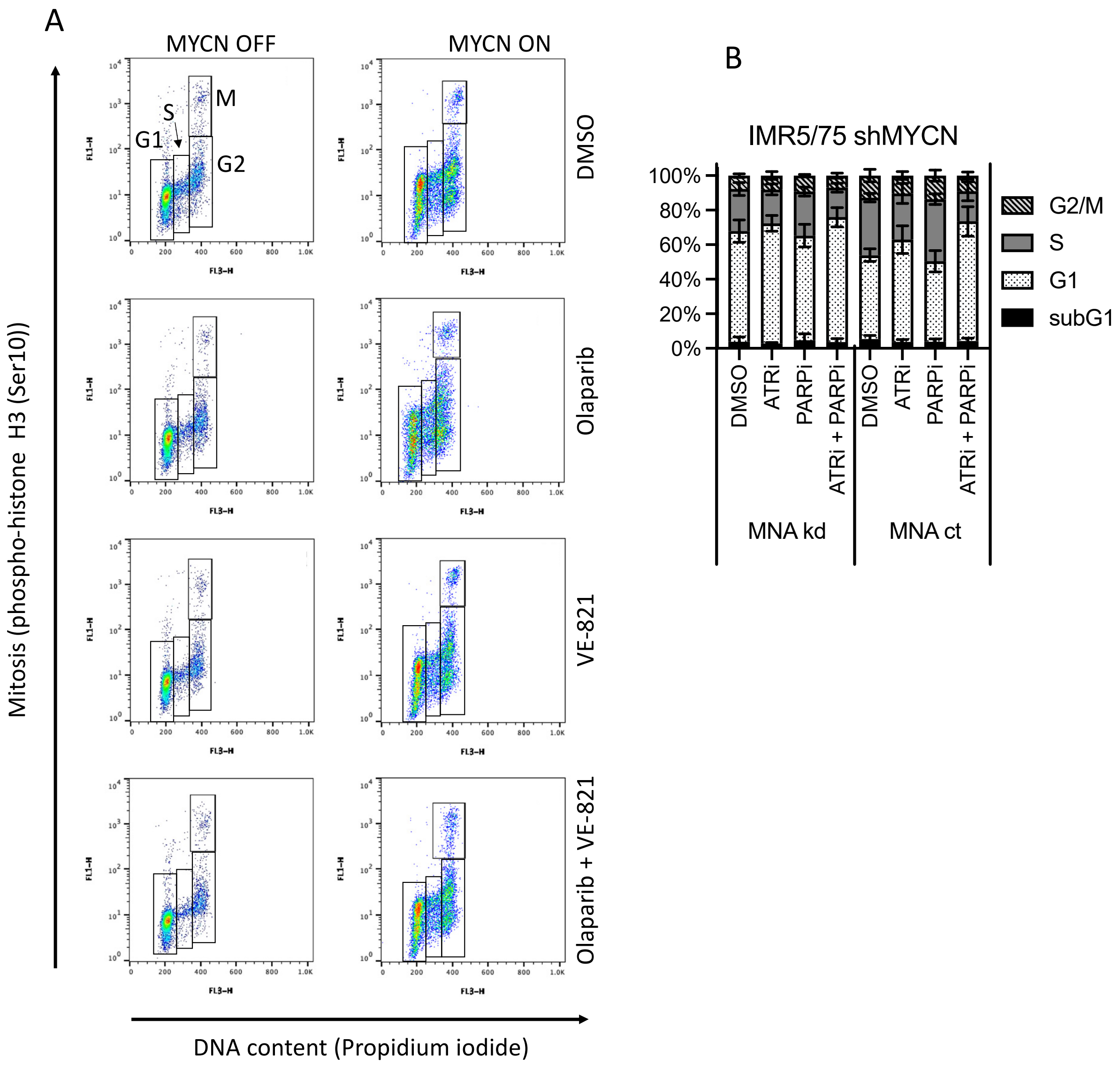

Figure S5. FACS plots for Figure 5 and cell cycle profile of IMR5/75 shMYCN cells. (A) example FACS plots from figure 5. (B) Cell cycle profile of IMR5/75 sh*MYCN* cells with *MYCN* amplified (MNA ct) and *MYCN* depleted (MNA kd) 24 hours post treatment 1 µM VE-821 and/or 1 µM olaparib or DMSO control, mean and standard deviation of 3 independent repeats each representing 10,000 cells.

MYCN OFF p-chk1 +VE	ND	1672993	ND	ND	ND	ND
MYCN OIFF chk1 + VE	2705546	3691538	2548504	2739937	2195865	2030561
MYCN OFF MYCN + VE	ND	ND	ND	ND	ND	ND
MYCN OFF ACTIN + VE	2367342	1823339	2106128	1777514	2098468	1355213
MYCN ON p-chk1 +VE	ND	1065499	ND	134799	ND	ND
MYCN ON Chk1 + VE	1324170	1068870	834501	726283	545879	231122
MYCN ON MYCN + VE	1863126	2002883	1364588	1206221	1217734	817001
MYCN ON ACTIN + VE	1323779	1728737	1823126	1762872	1847309	1042227

MYCN OFF p-chk1 +AZ	ND	1357800	ND	551652	ND	101270
MYCN OIFF chk1 + AZ	487090	504999	927457	1245694	1196313	1052539
MYCN OFF MYCN + AZ	ND	ND	ND	ND	ND	ND
MYCN OFF ACTIN + AZ	9163116	8842027	9157565	9635993	9023314	8884913
MYCN ON p-chk1 +AZ	ND	869461	ND	147331	ND	ND
MYCN ON Chk1 + AZ	2706356	2530077	2882029	2618859	2700359	2119954
MYCN ON MYCN + AZ	5548888	4558155	6545233	4771557	8024060	5927217
MYCN ON ACTIN + AZ	5775760	7394263	5110890	4544484	4213908	4804538

Figure S6. Full raw WBs for Figure 1. Quantification of bands seen in Figure 1: integrated density – background value (ND = not detected or below background) and Full western blots for Figure 1.

MYCN OFF p-chk1 +VE821

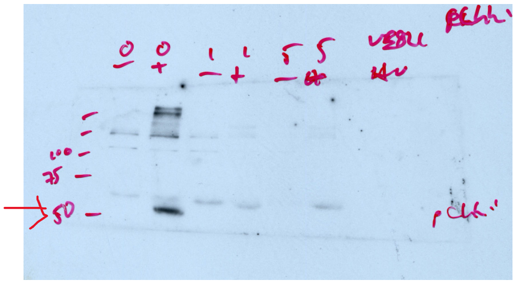

MYCN ON p-chk1 +VE821

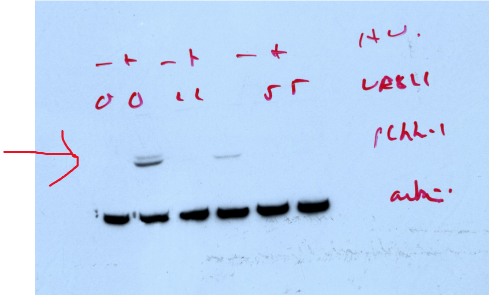

MYCN OFF chk1 +VE821

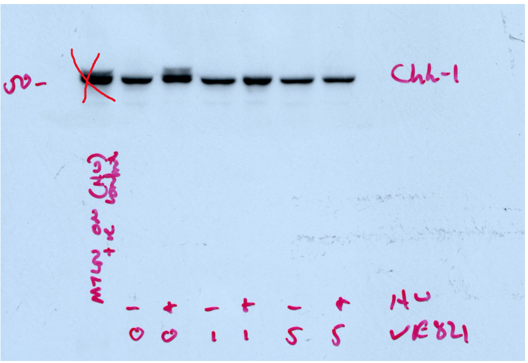

MYCN ON chk1 +VE821

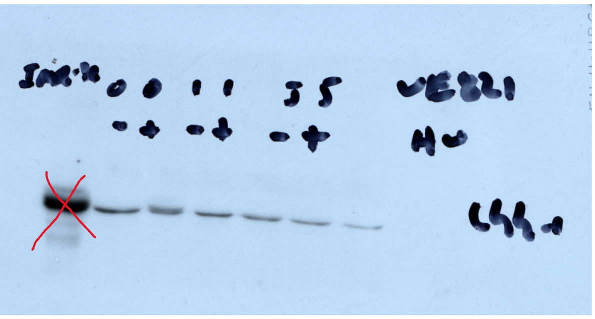

MYCN OFF MYCN +VE821

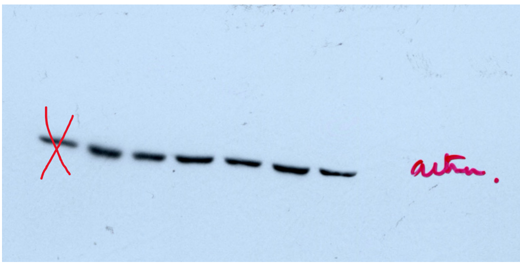

MYCN ON MYCN +VE821

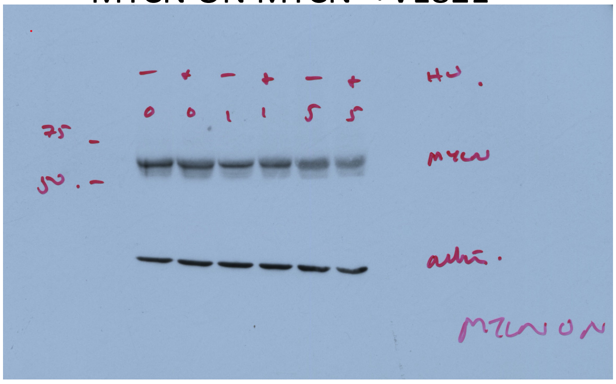

Figure S6. continued: Quantification of bands seen in Figure 1: integrated density – background value (ND = not detected or below background) and Full western blots for Figure 1.

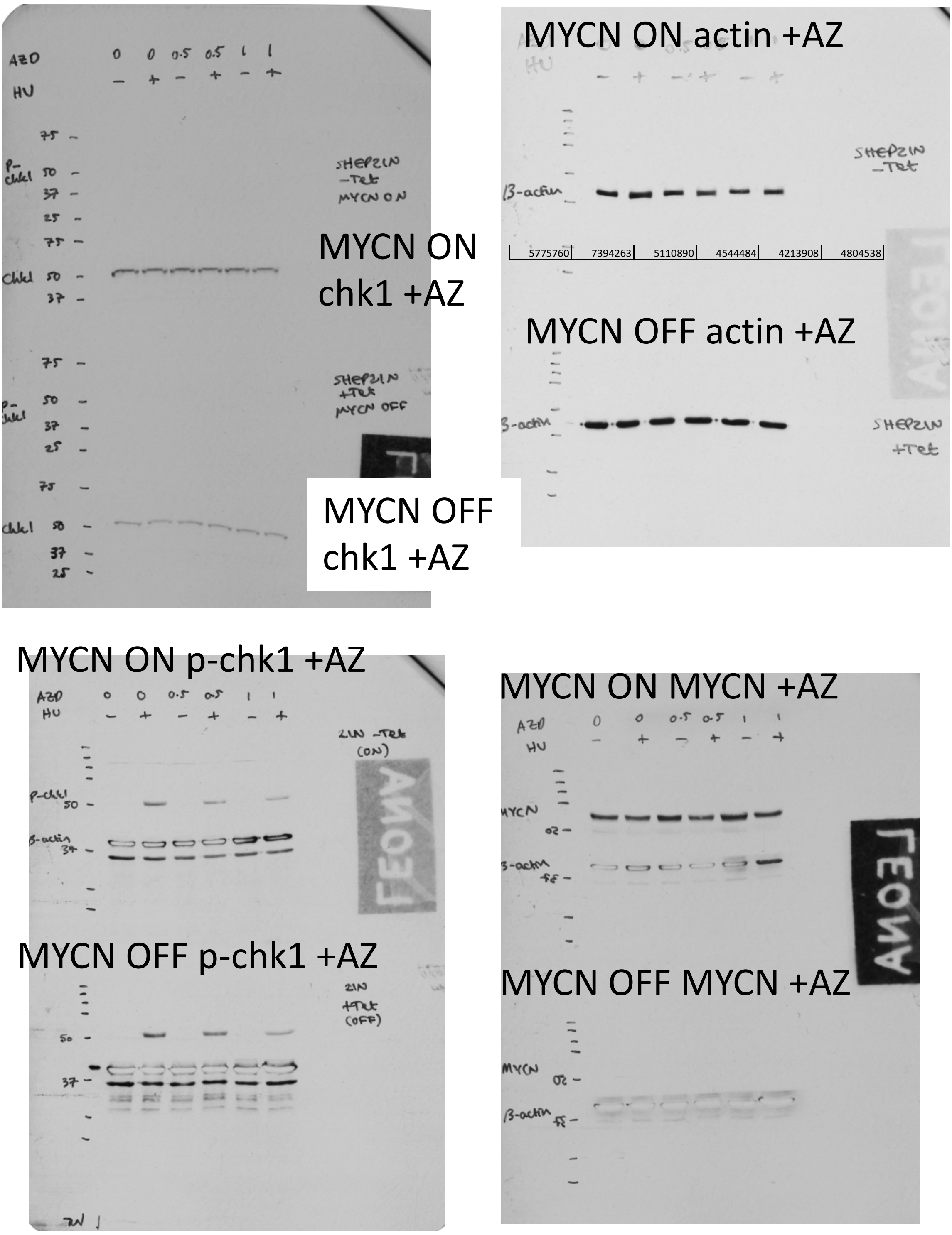

Figure S6. continued: Quantification of bands seen in Figure 1: integrated density – background value (ND = not detected or below background) and Full western blots for Figure 1.
